# Polymorphism in Gene for ABCC2 Transporter Predicts Methotrexate Drug Survival in Patients with Psoriasis

**DOI:** 10.3390/medicina57101050

**Published:** 2021-10-01

**Authors:** Jasna Grželj, Maruška Marovt, Pij B. Marko, Irena Mlinarič-Raščan, Tanja Gmeiner, Alenka Šmid

**Affiliations:** 1 Faculty of Pharmacy, University of Ljubljana, Aškerčeva cesta 7, 1000 Ljubljana, Slovenia; jg4288@student.uni-lj.si (J.G.); Irena.Mlinaric@ffa.uni-lj.si (I.M.-R.); tanja.gmeiner@ffa.uni-lj.si (T.G.); 2 Krka, d. d., Novo Mesto, Šmarješka cesta 6, 8000 Novo Mesto, Slovenia; 3 Department of Dermatovenerology, University Medical Centre Maribor, Ljubljanska ulica 5, 2000 Maribor, Slovenia; maruska.marovt@gmail.com (M.M.); pij.marko@ukc-mb.si (P.B.M.)

**Keywords:** psoriasis, drug survival, methotrexate, pharmacogenetics

## Abstract

*Background and Objectives*: Methotrexate is widely prescribed for the treatment of moderate-to-severe psoriasis. As drug survival encompasses efficacy, safety, and treatment satisfaction, such studies provide insights into successful drug treatments in the real-life scenario. The objective was to define methotrexate drug survival and reasons for discontinuation, along with factors associated with drug survival, in a cohort of adult patients with moderate-to-severe plaque psoriasis. *Materials and Methods*: Data on methotrexate treatment were extracted from our institutional registry. Drug survival was estimated by Kaplan–Meier analysis, and predictors of drug survival were analyzed by Cox proportional hazards regression. *Results*: We included 133 patients treated with methotrexate. Due to significant effects of the year of treatment initiation, drug survival analysis was performed for 117 patients who started methotrexate in 2010 or later. Median methotrexate drug survival was 11.0 months. Overall, 89% of patients discontinued treatment, with over half of these (51%) due to lack of efficacy. Significantly longer drug survival was seen for patients who discontinued treatment due to lack of efficacy versus drug safety (*p* = 0.049); when stratified by sex, this remained significant only for women (*p* = 0.002). The patient *ABCC2* rs717620 genotype was significantly associated with drug survival in both univariate log-rank and multivariate Cox regression analyses, with variant T allele associated with longer drug survival (hazard ratio, 0.606; 95% confidence interval, 0.380–0.967; *p* = 0.036). *Conclusions*: We have identified the novel association of patient *ABCC2* rs717620 genotype with methotrexate drug survival. This pharmacogenetic marker might thus help in the management of psoriasis patients in daily practice.

## 1. Introduction

Methotrexate is an immunosuppressive and antiproliferative agent with a well-established place in the treatment of patients with psoriasis [1,2]. It is the most commonly used conventional systemic drug and remains a first-line treatment for patients with moderate-to-severe psoriasis [2,3,4].

The chronic nature of psoriasis implies that patients require long-term treatment. Methotrexate is often used for maintenance therapy, although long-term treatment outcomes are rarely reported [5]. Furthermore, data from short-duration clinical trials performed with pre-selected patients need to be supported by data on methotrexate use in daily practice, e.g., from drug survival studies [6,7]. These studies are considered as real-life measures of treatment success, as they reflect drug efficacy and safety and indicate patient and physician preference [8,9]. Several studies have described drug survival of biologics [9], although data on methotrexate remain limited, even though methotrexate is still prescribed routinely as a first-line systemic agent and often represents a stepping-stone toward the introduction of biologics [7,10]. Therefore, an improved understanding of methotrexate treatment success in daily practice and the factors that impact upon methotrexate drug survival should contribute further to successful patient management.

Few studies have examined methotrexate drug survival in adult patients with psoriasis, with most comparing it to other conventional [10,11,12,13,14] or biological [10,11,12,14,15] agents. Four studies extensively focused on methotrexate [13,16,17,18], three of which looked for predictors of drug survival [13,16,18]. None of these investigated the possible effects of pharmacogenetic factors. Indeed, genetic factors might contribute to the marked variability of methotrexate treatment outcomes in psoriasis [2]. Most pharmacogenetic research of methotrexate in patients with psoriasis has investigated the polymorphisms in genes coding for proteins involved in the pharmacokinetic or pharmacodynamic pathways of methotrexate, with the latter predominantly focusing on enzymes of the folate cycle and the adenosine signaling pathway [2,19,20,21,22,23]. In our previous research, we used a hypothesis-driven approach for the selection of 16 candidate polymorphisms previously shown to affect the response to methotrexate or its pharmacokinetics or to be important for folate–homocysteine metabolism. Of these, five potential biomarkers of methotrexate treatment outcomes were identified: polymorphisms in genes coding for enzymes of the methionine cycle GNMT, DNMT3b, and BHMT (the first two were associated with methotrexate efficacy and the third with hepatotoxicity) and polymorphisms in genes for transporters ABCC2 and SLCO1B1 (both associated with efficacy) [24]. In this study we hypothesized that these pharmacogenetic markers also impact methotrexate drug survival.

The aim of this study was to define methotrexate drug survival and the reasons for discontinuation in a daily practice cohort of adult patients with moderate-to-severe plaque psoriasis. Furthermore, we investigated the factors associated with methotrexate drug survival, with a focus on pharmacogenetic markers.

## 2. Materials and Methods

### 2.1. Patients and Data Collection

Patients were recruited within a project for the establishment of an institutional registry of systemic treatment of adults with moderate-to-severe psoriasis, within which a methotrexate pharmacogenetics study was performed [24]. This study was approved by the National Medical Ethics Committee of Slovenia (No. 85/06/15) and performed according to the Declaration of Helsinki. Written informed consent was obtained from all patients who participated.

This retrospective study of methotrexate drug survival included 133 patients with moderate-to-severe plaque psoriasis treated with methotrexate as the only systemic agent (concomitant topical treatment was allowed) and for whom exact data on methotrexate treatment duration were available. Patient clinical characteristics and details of their methotrexate treatment were retrospectively collected from patient records and included age at introduction, start and stop dates, and reasons for treatment discontinuation.

Treatment discontinuation was defined as the cessation of treatment that resulted in a change of therapy (e.g., methotrexate with another conventional systemic drug, switch to a different systemic agent). By definition, drug survival is the time for which a patient continues a drug treatment [8], which was here calculated as the time from methotrexate start to when the event (methotrexate discontinuation) occurred. The reasons for discontinuation were classified into three categories: lack of efficacy; adverse events (AEs); and other reasons (as documented in patient records by treating physicians). Lack of methotrexate efficacy was defined as either inadequate response (i.e., Psoriasis Area and Severity Index (PASI) 75 not reached within 6 months) or when the patient records contained another explicit record of unsuccessful treatment or loss of efficacy beyond 6 months of treatment; both of these had to lead to methotrexate discontinuation and a change in therapy. The patients were stratified according to the year of methotrexate introduction, to examine changes in prescribing practice. As 2010 was the first full year of ustekinumab availability, it was considered the most appropriate cut-off for these patients.

### 2.2. Determinants of Drug Survival

Patient characteristics in terms of their temporal relationship to methotrexate treatment were defined as possible determinants of drug survival. Furthermore, previously identified pharmacogenetic markers of methotrexate treatment outcomes [24] were also considered. Six single nucleotide polymorphisms (SNPs) in five genes encoding enzymes of the methionine cycle or methotrexate transporters were included in the analysis; they are listed in Section 2.3.

### 2.3. DNA Extraction and Genotyping

DNA was extracted from patient venous blood samples using the MasterPure Complete DNA and RNA purification kits (Epicentre, Illumina, Madison, WI, USA) or QIAamp DNA Mini kits (Qiagen, Hilden, Germany) as per the manufacturers’ instructions. Genotyping was performed by means of TaqMan genotyping assays (Applied Biosystems, Foster City, CA, USA) using the Roche LightCycler 480 system, in line with manufacturer instructions. The following assays were used for analysis of selected SNPs: TaqMan assay ID C__11646606_20 (rs3733890, *betaine-homocysteine methyltransferase*; *BHMT*); assay ID C__11425842_10 (rs10948059, *glycine N-methyltransferase*; *GNMT*); assay ID C__25620192_20 (rs2424913, *DNA [cytosine-5-]-methyltransferase 3β*; *DNMT3b*); assay ID C___2814642_10 (rs717620, *ATP-binding cassette transporter C2*; *ABCC2*); assay IDs C___1901697_20 and C__30633906_10 (rs2306283 and rs4149056, respectively, *solute carrier organic anion transporter family member 1B1*; *SLCO1B1*).

### 2.4. Statistical Analysis

Descriptive statistics were used for the characteristics of the study cohort. Continuous variables are reported as means ± standard deviation (SD) if parametric and medians ± ranges if non-parametric. Counts and percentages were calculated for categorical data.

For the analysis of genotype associations, patients were divided into two groups using the dominant model (wild-type vs. heterozygous/homozygous genotype). The exception was *SLCO1B1*, where two groups were formed according to reported transporter activity associated with established haplotypes: (i) high-activity group comprising of haplotypes SLCO1B1*1a (wild-type) and SLCO1B1*1b (variant rs2306238) and (ii) low-activity group including patients with haplotypes SLCO1B1*5 (variant rs4149056) and SLCO1B1*15 (variant for both rs2306238 and rs4149056).

Drug survival was investigated using Kaplan–Meier survival analysis. Observations were censored either at the time of data-lock if no discontinuation was seen or for patients lost to follow-up. Log-rank tests examined the differences in drug survival for the reasons for discontinuation and selected factors, including sex, age at methotrexate introduction, age at psoriasis onset, family history, and the six genotypes previously identified as significant for methotrexate treatment outcomes. The effects of changing prescription practices were investigated using log-rank tests for the year of methotrexate introduction (pre-2010/2010 and later).

Multivariate Cox proportional hazards regression with forward/backward selection was performed to identify the predictors of drug discontinuation. All variables tested by log-rank tests were entered into the model, noting that the number of variables included in multivariate Cox regression models should not exceed 10% of the observed events [8]. Hazard ratios (HRs) with 95% confidence intervals (CIs) were calculated to describe the risks associated with the analyzed factors. The level of significance was set at α = 0.05. All analyses were performed using IBM SPSS version 25.0 (IMB Corp., Armonk, NY, USA).

## 3. Results

### 3.1. Patient and Treatment Characteristics

Out of 133 included patients, 81 (60.9%) were male. The mean age at psoriasis onset was 29.9 ± 14.4 years, and 102 patients (76.7%) showed early onset psoriasis (diagnosis before 40 years [20]). Information on family history of psoriasis was available for 131 patients and was positive in 64 (48.9%) individuals. Genotypes for six candidate polymorphisms in five genes were determined for the included patients. Variant allele frequencies are presented in Table 1.

All patients were initially treated with 15 mg methotrexate weekly, with concomitant folic acid (5 mg weekly, 24 h post-methotrexate). At methotrexate introduction, the mean age was 49.9 ± 12.7 years, with median disease duration 17.0 years (range 0.00–66.0 years). The median methotrexate treatment duration was 11.1 months (range 0.92–202.1 months).

Most patients discontinued methotrexate treatment during the period of analysis. Only 14 patients (10.5%) still received methotrexate at data-lock. Most of the 119 patients who discontinued treatment (*n* = 62; 52.1%) did so for lack of efficacy, while 41 patients (34.5%) experienced AEs that required treatment cessation. The remaining 16 patients (8.04%) discontinued treatment for other reasons.

### 3.2. Change in Prescription Practice: Influence of Time of Methotrexate Introduction

Patient inclusion was not restricted by year of therapy initiation. Methotrexate introduction ranged from May 1999 to September 2016, during which time the psoriasis treatment landscape changed drastically. As the availability of treatment alternatives might influence drug survival [8,25], patients were classified according to the year of treatment introduction, with the first full year of ustekinumab availability (i.e., 2010) as the cut-off. Of the 133 patients, 16 (12.0%) started treatment before 2010.

To test our assumption of an influence of the introduction year on methotrexate drug survival, the two groups (pre-2010 vs. 2010 and later) were compared using log-rank tests. A significant difference (*p* = 0.005) was identified, whereby patients who started methotrexate therapy before 2010 continued treatment for longer (median drug survival, 36.0 months; 95% CI, 0.00–110.4 months) compared to patients who started methotrexate treatment in 2010 or later (11.0 months; 95% CI, 7.97–14.0 months).

### 3.3. Methotrexate Drug Survival in Patients Starting Therapy in 2010 or Later

Due to the identified influence of the year of treatment introduction, the analysis of methotrexate drug survival using Kaplan–Meier survival analysis was conducted only for the subpopulation of patients who started methotrexate treatment in 2010 or later (*n* = 117). Their patient and treatment characteristics are shown in Table 2.

**Figure 1:**
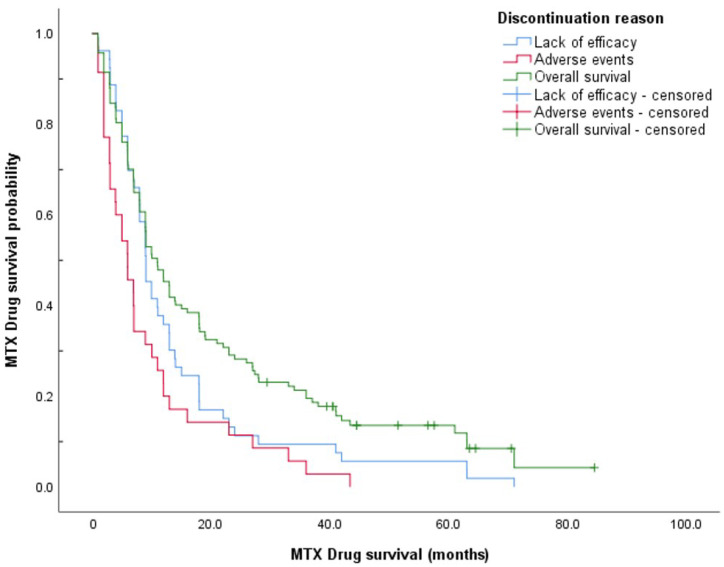
Methotrexate drug survival curves according to reason for discontinuation.

The overall median methotrexate drug survival was 11.0 months (95% CI, 7.97–14.0 months). After 1 year of methotrexate treatment, 46.2% of patients were still on the therapy; the drug survival rate then dropped to 28.2% at 2 years of methotrexate treatment. Only 6.8% of patients reached 5 years of methotrexate treatment. Patients who discontinued methotrexate treatment due to lack of efficacy showed longer drug survival than those who experienced AEs: median drug survival, 8.02 months (95% CI, 6.37–9.67 months) versus 5.98 months (95% CI, 3.12–8.84 months), respectively. Figure 1 shows Kaplan–Meier survival curves for overall drug survival and according to reasons for discontinuation.

Survival curves of the two discontinuation groups (i.e., lack of efficacy vs. AEs) were compared using log-rank tests, with a significant difference seen (*p* = 0.049). To gain further insight into this finding, the groups were stratified by sex. This difference in drug survival according to the reason for discontinuation appears to be solely driven by the significant difference in survival curves for females (log-rank test, *p* = 0.002), as this was lost for males (*p* > 0.05). Median drug survival for women discontinuing methotrexate treatment for lack of efficacy was 9.04 months (95% CI, 6.02–12.1 months) compared to 3.02 months (95% CI, 0.96–5.08 months) for AEs.

**Figure 2:**
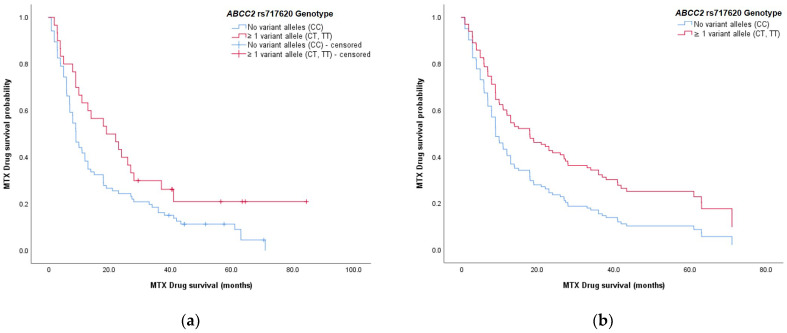
Methotrexate drug survival curves stratified according to the *ABCC2* rs717620 genotype: (**a**) drug survival curves obtained by Kaplan–Meier survival analysis; (**b**) theoretically predicted survival curves obtained by multivariate Cox regression analysis. Both of the survival plots show a higher probability of longer drug survival for patients who carry at least one variant allele T in *ABCC2* (red), compared to the wild-type genotype (blue).

Further analyses using log-rank tests determined whether selected categorical determinants led to differences in methotrexate drug survival. A significant difference in survival curves was seen when patients were split according to the *ABCC2* rs717620 genotype (*p* = 0.030) (Figure 2). At least one copy of variant allele T (-24CT, -24TT genotypes) was associated with longer median drug survival compared to the wild-type (-24CC) genotype: 19.1 months (95% CI, 7.02–31.1 months) versus 9.04 months (95% CI, 7.47–10.6 months), respectively. No significant differences were identified for patient sex, family history, *BHMT* rs3733890, *GNMT* rs10948059, and *DNMT3b* rs2424913 genotypes, and *SLCO1B1* haplotype (rs2306238/rs4149056).

### 3.4. Predictors of Drug Survival

To search for the determinants of drug survival, a multivariate analysis was performed using Cox proportional hazards regression with forward and backward variable selection. The selection model included all variables tested in the Kaplan–Meier drug survival analysis with log-rank tests (i.e., sex; *BHMT*, *GNMT*, *DNMT3b*, and *ABCC2* genotypes; *SLCO1B1* haplotype) and numerical variables (age at methotrexate introduction, age at disease onset). As family history correlates with age at disease onset, this was left out of the model.

The *ABCC2* genotype was significantly associated with methotrexate drug survival (Table 3). Patients carrying at least one variant allele T (-24CT, -24TT genotypes) had a lower hazard ratio for treatment discontinuation (HR, 0.606; 95% CI, 0.380–0.967; *p* = 0.036). Therefore, they had a greater chance of longer methotrexate use compared to patients with the wild-type-24CC *ABCC2* rs717620 genotype; this was in line with the univariate log-rank tests (Figure 2).

None of the other factors in the multivariate model were significantly associated with drug survival. Results from the univariate and multivariate analyses are given in Table 3.

## 4. Discussion

Methotrexate is widely used for treatment of moderate-to-severe psoriasis. It is the most frequently prescribed conventional systemic agent, and its use commonly precedes biologic therapy [7,10]. Treatment outcomes for methotrexate vary widely among patients, and many discontinue methotrexate due to a lack of efficacy or the occurrence of AEs [2]. Despite the frequent use of methotrexate, real-life data on its efficacy, safety, and duration of therapy remain lacking [10,16]. Drug survival studies provide such data and can offer insight into the therapeutic success of methotrexate in daily practice.

In the present study, we described methotrexate drug survival in a cohort of 133 patients with moderate-to-severe plaque psoriasis. We recorded a median methotrexate drug survival of 11.1 months. Almost 90% of patients discontinued treatment during the observation time, predominantly due to a lack of efficacy. We identified a significant impact of the year of methotrexate introduction on drug survival, and although we divided the patients into only two groups according to the entry of ustekinumab onto the market, a pronounced reduction in the median methotrexate drug survival was observed. This is in line with the suggested impact of changing prescription practices and the reported effects of increased biologics availability, which resulted in a shorter treatment duration of both conventional and biologic agents [8,13,25].

To prevent confounding by the changes in the psoriasis management landscape, we performed drug survival analyses on the group of 117 patients who started methotrexate treatment in 2010 or later. Almost the same proportion of patients discontinued treatment in this group, with a similar median methotrexate drug survival of 11.0 months. Other studies that have investigated methotrexate drug survival in adult patients with psoriasis have reported median drug survivals of 9.15 months [26], 12.1 months [11], 15 months [18], 18 months [13], 21.6 months [16], and 29.3 months [15]. Additionally, mean drug survivals have been reported as 7.7 months [14], 17.2 months [27], 18.8 months [17], and 22.3 months [10]. The median methotrexate drug survival in the present study thus lies on the low end of this spectrum, as half of the patients included had discontinued treatment after 11 months. However, differences in the studies performed make comparisons difficult to interpret, such as the inclusion of patients with different psoriasis types [10,11,12,15,26] versus plaque psoriasis only [14,16,18] and uncertainty for the combination therapy or concomitant folic acid [10,11,12,13,14,15,16,26]. For these aspects, the present cohort was similar to that of Otero and colleagues [16], where patients with plaque psoriasis were prescribed methotrexate monotherapy and folic acid supplementation. Despite this, the median methotrexate drug survival in the present study was almost half that of Otero and colleagues [16]. This might have arisen from a difference in prescription guidelines and also, importantly, in reimbursement practices [7,10,28].

Almost 90% of patients in the present study discontinued methotrexate treatment during the observation period. A lack of efficacy was the main reason given, with a little over half of the treatment cessations (51.0% of discontinuations in the subgroup of patients starting methotrexate in 2010 or later). This was a surprise finding, as AEs were expected to be the main driver of therapy cessation, in line with existing data [10,11,16,17,18]. Furthermore, at 51%, the rate of discontinuation due to a lack of efficacy in the present study was significantly greater than that for other methotrexate drug survival studies, which ranged from 16% to 33% [10,11,12,16,17,18,29]. In contrast, the proportion of patients who stopped methotrexate therapy due to AEs was relatively consistent with previously reported data (present study: 34%; other studies: 12–47% [10,11,12,16,17,18,29]).

Interestingly, patients who discontinued methotrexate due to a lack of efficacy did so with a longer drug survival than their counterparts who experienced AEs, with significantly different drug survival curves between these two groups. Moreover, when we stratified the data according to sex, there was a pronounced difference for women: the median methotrexate drug survival for women who discontinued due to a lack of efficacy was three-fold that for AEs, with this relationship lost for men. The women in this cohort did not experience more AEs or report them more often; however, these data suggested that when women did experience AEs, methotrexate therapy was more readily discontinued. As far as we know, differences in the discontinuation of methotrexate according to sex for patients with psoriasis have not been reported previously; further, studies of methotrexate in rheumatoid and psoriatic arthritis have not identified sex as a determinant for drug survival [30,31]. In addition, we note that our overall drug survival analysis using both Kaplan–Meier analysis and Cox regression analysis did not identify sex as a factor that influenced methotrexate drug survival. Separate analyses according to reasons for discontinuation would have been needed to confirm this influence of sex, but these were not performed due to the limited sample size and the ensuing concerns of statistical power. However, these findings warrant further exploration.

To the best of our knowledge, this is the first study to date to investigate the effects of genetic polymorphisms on methotrexate drug survival in patients with psoriasis. Here, we identified the *ABCC2* rs717620 genotype as a determinant of methotrexate drug survival in plaque psoriasis in both the univariate analysis using log-rank tests and the multivariate Cox proportional hazards regression. The variant allele conveyed a significant protective effect (HR, 0.606), as patients carrying at least one variant allele T (i.e., -24CT, -24TT) achieved longer drug survival compared to patients with wild-type -24CC genotype. ABCC2 is a transporter that is important for the efflux of natural folates and antifolate drugs from cells [32]. The common polymorphism rs717620 has been associated with reduced transporter activity, which leads to decreased methotrexate elimination and hence higher methotrexate exposure [33,34,35,36]. Our previous study showed an important association between *ABCC2* rs717620 genotype and methotrexate efficacy in psoriasis, whereby variant allele carriers were more likely to achieve adequate disease control with methotrexate [24], possibly due to this increased methotrexate exposure. The impact of *ABCC2* rs717620 polymorphism on methotrexate drug survival reported here is in line with these previous data [24], whereby patients carrying variant allele T were more likely to continue treatment for longer, implying longer duration of disease control. Treatment response itself was associated with rs717620 polymorphism in the same way, i.e., variant allele T increased the odds of adequate treatment response [24]. Interestingly, considering the polymorphisms that we previously identified as significant for methotrexate efficacy and safety, the polymorphism in the ABCC2 transporter gene was the only one to also have effects here on methotrexate drug survival. Polymorphisms in genes for the transporter SLCO1B1 and the methionine cycle enzymes GNMT, DNMT3b, and BHMT did not have any effects on methotrexate drug survival in the present study, even though some of these had shown strong associations with methotrexate treatment outcomes in our previous study [24].

Only a handful of studies have investigated predictors of methotrexate drug survival to date. These have identified only isolated factors, without consistency among studies. Age at methotrexate introduction was identified as significant in two studies [13,18], but not in a third [16], and now not in the present study. Age at psoriasis onset was also not identified as a significant factor here, in line with previous results [16]. Concomitant metabolic syndrome, oral administration, and folic acid supplementation were identified as protective factors by Shalom et al. [13] which was also the only study to examine these factors [13]. As all patients in our cohort received methotrexate orally and were supplemented with folic acid, such an analysis was not feasible here. A positive association of ≥15 mg methotrexate weekly and drug survival has also been reported [18], in line with the known dose–response effects of methotrexate [2]. Otero et al. [16] reported no association of baseline disease severity and overall survival in multivariate analyses; however, severity perception by patients predicted short drug survival when methotrexate was discontinued due to AEs [16]. We did not include severity variables in the present analysis; although we assessed severity by PASI, Body Surface Area, and/or the Dermatology Life Quality Index, none of these were used for all or the majority of patients. Interestingly, neither the present study nor other studies have identified sex as an important factor for drug survival in multivariate Cox analyses [13,16,18], which contrasts with the results for biologic treatments [9]. Overall, at this stage, the determinants of methotrexate drug survival identified to date need further replication before they can lead to any meaningful applications in clinical practice.

An interesting finding from the present study was the identification of a novel genetic factor associated with methotrexate drug survival—a result that supplements a previously reported association with methotrexate treatment outcome. It therefore appears that *ABCC2* rs717620 has additional potential as a biomarker of methotrexate treatment in adults with moderate-to-severe psoriasis.

Some limitations of the present study should be acknowledged when interpreting these findings. First, we gathered data retrospectively, which precluded the collection of some patient or disease characteristics (e.g., smoking at the time of treatment, disease severity using the same standardized criteria in all patients). Second, the observational nature of the study carries a risk of bias inherent to this type of study; confounding and selection bias cannot be fully excluded as sources of error. Furthermore, we included a limited number of patients, and thus it was not feasible to perform separate multivariate analyses according to reasons for discontinuation. Of note, approximately one-third of Slovenian patients with moderate-to-severe psoriasis are treated at our institution (and virtually all consented to inclusion in the patient registry); hence our cohort can be considered as representative for the country. Despite this, we should also acknowledge that the influence of the pharmacogenetic marker identified in this study should be considered as preliminary, as no definite conclusions can be made due to the small sample size. Our findings hence need further validation in larger patient cohorts, if possible, in prospective studies.

## 5. Conclusions

This study describes methotrexate treatment in a daily practice cohort of patients with moderate-to-severe psoriasis. To the best of our knowledge, this is the first study that has investigated the effects of genetic predisposition on methotrexate drug survival. These data show that genetic variability in the gene coding for the ABCC2 transporter influences methotrexate drug survival, whereby a patient carrying at least one copy of variant *ABCC2* rs717620 allele is likely to remain on treatment for longer compared to patients with wild-type *ABCC2* rs717620. Our findings suggest pharmacogenetic markers might have further applications in the management of moderate-to-severe psoriasis and might represent an interesting addition to studies of methotrexate use in daily practice.

## Figures and Tables

**Table 1:** Genotype data and variant allele frequencies of the analyzed polymorphisms in included patients.

Gene	Variant	Wild-Type (*n*)	Heterozygous (*n*)	Homozygous (*n*)	Variant Allele Frequency (%)
*BHMT*	rs3733890	68	50	15	0.301
*GNMT*	rs10948059	36	62	35	0.496
*DNMT3b*	rs2424913	38	60	35	0.489
*ABCC2*	rs717620	95	30	7	0.167
*SLCO1B1* *	rs2306283	40	81	11	0.390
*SLCO1B1* *	rs4149056	84	45	2	0.187

* Haplotypes based on the genotype combinations for both polymorphisms, classified according to activity: high-activity haplotypes SLCO1B1*1a, *n* = 30 and SLCO1B1*1b, *n* = 54; low-activity haplotypes SLCO1B1*5, *n* = 10 and SLCO1B1*15, *n* = 37. *ABCC2, ATP-binding cassette transporter C2*; *BHMT*, *betaine-homocysteine methyltransferase*; *DNMT3b*, *DNA (cytosine-5-)-methyltransferase 3β*; *GNMT*, *glycine N-methyltransferase*; *SLCO1B1*, *solute carrier organic anion transporter family member 1B1.*

**Table 2:** Patient and treatment characteristics of the subpopulation of patients with methotrexate introduction in 2010 or later.

Characteristic	Patients Starting Treatment in 2010 or Later (*n* = 117)
Male (*n* (%))	71 (60.7)
Mean age at disease onset (years (SD))	29.2 (14.5)
Mean age at MTX introduction (years (SD)	50.2 (12.9)
Early onset psoriasis ^1^ (*n* (%))	91 (77.8)
Positive family history (*n* (%))	60 (51.7)
Median disease duration at MTX introduction (years (range))	20.0 (0.00–66.0)
Median duration of MTX treatment (months (range)	11.0 (0.99−84.5)
Discontinued treatment (*n* (%))	104 (88.9)
- Lack of efficacy	53 (51.0)
- Adverse events	35 (33.7)
- Other	16 (15.4)

^1^ Onset at or before the age of 40 years. MTX, methotrexate.

**Table 3:** Determinants of methotrexate drug survival from the results of the univariate tests and multivariate analyses.

Factor	Detail	Patients (*n*)	Events (*n* (%Patients))	Univariate Analysis (Log-Ranks)	Multivariate Analysis (Cox Regression) *	
				*p*-Value	Hazard Ratio (95% CI)	*p*-Value
Sex	Male (Ref)	71	63 (88.7)	0.487		
	Female	46	41 (89.1)			
Family history	Negative	56	50 (89.3)	0.592	Not included	
	Positive	60	53 (88.3)			
*BHMT* genotype	GG (Ref]	57	49 (86.0)	0.203		
	GA or AA	60	55 (91.7)			
*GNMT* genotype	CC (Ref)	30	25 (83.3)	0.750		
	CT or TT	87	79 (90.8)			
*DNMT3b* genotype	CC (Ref]	34	28 (82.4)	0.630		
	CT or TT	83	76 (91.6)			
*ABCC2* genotype	CC (Ref)	86	80 (93.0)	**0.030**	0.606 (0.380–0.967)	**0.036**
	CT or TT	30	23 (76.7)			
*SLCO1B1* haplotype activity	High activity (Ref)	74	67 (90.5)	0.216		
	Low activity	41	35 (85.4)			

* Adjusted for age at disease onset and age at methotrexate introduction. ABCC2, ATP-binding cassette transporter C2; BHMT, betaine-homocysteine methyltransferase; CI, confidence interval; DNMT3b, DNA (cytosine-5-)-methyltransferase 3β; GNMT, glycine N-methyltransferase; Ref, reference category in multivariate analysis (for genotypes, Ref. denotes wild-type genotype); SLCO1B1, solute carrier organic anion transporter family member 1B1. Significant data are given in bold.

## Data Availability

Due to patient privacy concerns, data used and/or analyzed during this study are not available for public access. Data are available from the corresponding author upon reasonable request.
